# Severe pyoderma gangrenosum caused by myelodysplastic syndrome successfully treated with decitabine administered by a noncytotoxic regimen

**DOI:** 10.1002/ccr3.1221

**Published:** 2017-10-31

**Authors:** Mostafa F. M. Saleh, Yogen Saunthararajah

**Affiliations:** ^1^ Department of Hematology and Oncology Taussig Cancer Institute Cleveland Clinic Cleveland Ohio; ^2^ Internal Medicine Department Assiut University Hospitals Assiut Egypt

**Keywords:** Decitabine, differentiation therapy, myelodysplastic syndrome, pyoderma gangrenosum

## Abstract

Pyoderma gangrenosum (PG) is a morbid necrotizing neutrophilic dermatoses for which current treatments are inadequate. Here, we describe the use of a novel noncytotoxic regimen of the deoxycytidine analog decitabine to treat underlying myeloid malignancy causing PG, to thereby produce safe and effective resolution of extensive PG.

Pyoderma gangrenosum (PG) is a neutrophilic, inflammatory dermatosis that produces necrotizing ulcers. PG begins with an erythematous nodule or hemorrhagic pustule that rapidly evolves into a necrotic, painful ulcer with an undermined border and surrounding induration/erythema. Lesions can be single or multiple. Most patients have an identifiable underlying systemic disease, typically inflammatory bowel disease (~65%), less often inflammatory arthiritis (~15%) or myeloid malignancies such as myeloproliferative neoplasms or myelodysplastic syndromes (MDS) (~12.5%) 1. In addition to severe painful morbidity, PG has been linked with an increased risk for mortality 2. Standard treatment is with corticosteroids and/or cyclosporine immune‐suppression, although healing of fewer than 50% of ulcers with prolonged treatment and of ~20% of ulcers within 6 weeks was noted in both arms of a prospective clinical trial that compared oral prednisone 0.75 mg/kg/day versus cyclosporine 4 mg/kg/day (*n* = 121) 3. Better treatments are thus needed, ideally based on effective treatment of underlying systemic disease.

A 50‐year‐old woman presented with a very large circumferential weeping ulcer of the left lower leg, evolved from “red bumps” that became increasingly painful, ulcerated, and coalesced over a 5‐month period. Other symptoms were night sweats and fevers (T^max^ 38.3°C). She had no symptoms to suggest inflammatory bowel disease or arthiritis, and the only risk factor for myeloid malignancy was a history of smoking (>30 pack years). On examination, there was an extensive crusted and weeping ulcer afflicting almost the entire lower left leg circumferentially from ankle to knee (Fig. 1A). A skin biopsy from the indurated ulcer margin demonstrated a diffuse infiltrate of the dermis by neutrophils and some lymphocytes. Anemia (hemoglobin 10 g/dL) and neutropenia (0.5 × 10^9^/L) prompted consideration of an underlying myeloid malignancy, confirmed by a bone marrow aspirate and biopsy examination showing hypercellular marrow (80%) with multi‐lineage dyspoiesis including atypical neutrophil segmentation and mild hypogranularity, without an increase in myeloblasts. Standard metaphase karyotyping has demonstrated clonal deletions of chromosome 9 (q22q34) and additions of chromosome 20 (q11.2) in 18 metaphases and deletion of chromosome 7 (q21q35) in 1 metaphase. Thus, she was diagnosed with PG caused by MDS. High‐dose intravenous corticosteroids (methylprednisolone) and immunoglobulin therapy was administered for 5 days followed by oral prednisone 1 mg/kg. Mild, transient improvement in the ulceration and resolution of anemia was followed by progressive ulceration over 12 weeks, while still on corticosteroids. A repeat bone marrow evaluation again demonstrated chromosome 9 and 20 alterations in 20 metaphases.

**Figure 1:**
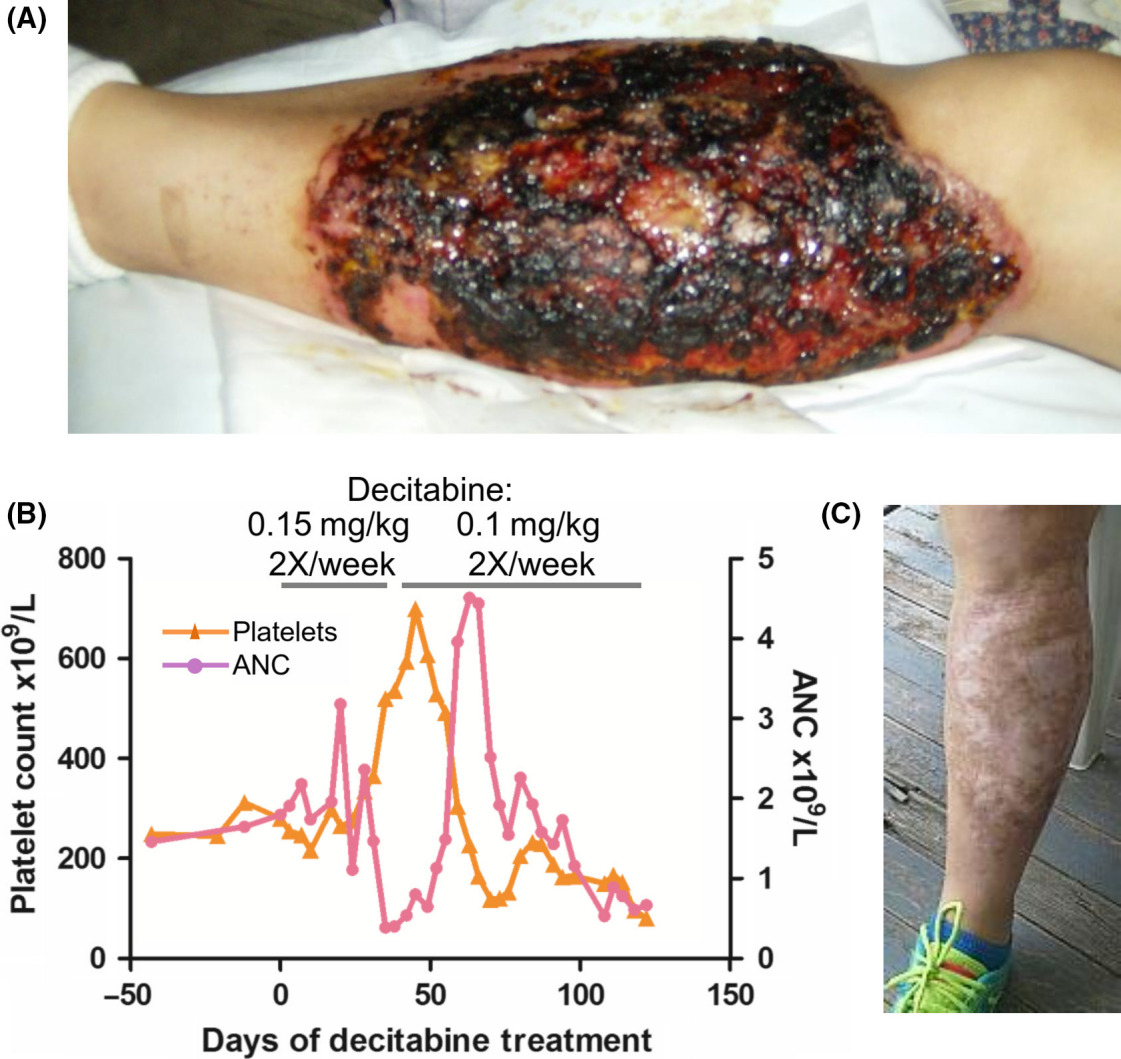
Successful treatment of severe PG caused by MDS by a noncytotoxic regimen of decitabine to deplete DNMT1. (A) The ulcer pretreatment. (B) Treatment induced increase in platelet count consistent with noncytotoxic differentiation‐restoring mode of action. (C) The ulcer after 20 weeks of therapy.

Decitabine is a DNA methyltransferase (DNMT1) depleting drug approved for treatment of MDS, but also has cytotoxic effects when administered by standard pulse‐cycled intravenous regimens. Difficult wound management and the need for wound healing thus deterred administration of standard regimens, and instead, decitabine was administered by a regimen designed to increase the epigenetic, terminal‐differentiation restoring therapeutic effect of DNMT1‐depletion while simultaneously avoiding cytotoxicity: Dose was reduced to 0.15 mg/kg/day compared to the FDA‐approved 20–45 mg/m^2^/day (a 75–90% reduction) to avoid cytotoxicity and these well‐tolerated doses were administered frequently 2X/week instead of instead of pulse‐cycled 3–5 days/4–6 weeks, to increase probabilities that malignant cell S‐phase entries would coincide with drug exposure, needed for S‐phase‐dependent DNMT1‐depletion 4. In the first 8 weeks, this treatment increased platelet counts and decreased neutrophils, shifts in hematopoietic differentiation expected from noncytotoxic DNMT1‐depletion of malignant clones 5 (Fig. 1B). By week 8, there was substantial decrease in pain and extensive granulation of previously ulcerated areas, permitting reduction in opiate and steroid dosage. Because of treatment‐induced neutropenia, the decitabine dose was reduced to 0.1 mg/kg 2X/week from week 9. By week 12, there was skin cover over >60% and granulation of the remaining previously ulcerated area. By week 20, the ulcer was completely healed, and prednisone therapy was tapered to off (Fig. 1). Nadirs in all blood lineages occurred between week 9 and 20, as cytoreduction of the malignant clone after terminal‐differentiation induction is expected also to decrease its contribution to peripheral blood counts, even as other normal dividing cells (e.g., skin layers) are not affected (Fig. 1) 5. Repeat bone marrow evaluation at week 20 confirmed cytoreduction of malignant hematopoiesis with hypocellularity (20%), mild erythroid dysplasia as the sole dysplasia, and normal cytogenetics (10 metaphases). She proceeded to stem cell transplant from an unrelated donor. A post‐transplant bone marrow aspirate and biopsy demonstrated 100% donor chimerism and normal cytogenetics in 20 metaphases. She has remained without evidence of disease >3 years post‐transplant.

Although MPN/MDS typically presents with abnormal peripheral blood counts, a well‐established though less typical mode of presentation is with clinically significant inflammation, such as interstitial pneumonitis, neutrophilic dermatoses (Sweet's disease, PG) that can be accompanied by systemic symptoms such as fever, night sweats, and weight loss. The pathophysiology underlying PG is not clearly understood; however, a well‐described element is neutrophil dysfunction (defects in chemotaxis, migration, phagocytosis), that can be a feature of quantitatively and qualitatively defective clonal hematopoiesis in myeloid malignancies. Skin biopsies in PG are not usually diagnostic or informative as to underlying systemic disease, and a diagnosis of a causative myeloid malignancy is based on evidence of clonal hematopoiesis, for example, dysplasia of blood cells in the peripheral blood or bone marrow, often accompanied by measurable clonal chromosomal or single base genetic alterations. Current standard treatment of MDS is with DNMT1‐depleting (‘hypomethylating’) drugs 5‐azacytidine or decitabine. There were no reports of these drugs successfully treating PG caused by myeloid malignancy. Here, we provide the first report of successful treatment of corticosteroid‐resistant PG caused by MDS by decitabine administered by a regimen designed to deplete DNMT1 without cytotoxicity 4. This noncytotoxic regimen has also been reported to resolve MDS‐associated Sweet's syndrome in two patients 4, 6. Thus, this approach can be used to successfully treat MDS and its associated neutrophilic dermatoses.

## Authorship

MS: collected data and contributed to the writing of the manuscript. YS: managed the patient and wrote the manuscript.

## Conflict of Interest

No potential financial conflicts of interest to disclose.
